# An Improved Composite Multiscale Fuzzy Entropy for Feature Extraction of MI-EEG

**DOI:** 10.3390/e22121356

**Published:** 2020-11-30

**Authors:** Mingai Li, Ruotu Wang, Dongqin Xu

**Affiliations:** 1 Faculty of Information Technology, Beijing University of Technology, Beijing 100124, China; s201602089@emails.bjut.edu.cn (R.W.); Xudq@emails.bjut.edu.cn (D.X.); 2 Beijing Key Laboratory of Computational Intelligence and Intelligent System, Beijing 100124, China; 3 Engineering Research Center of Digital Community, Ministry of Education, Beijing 100124, China

**Keywords:** weighted composite multiscale fuzzy entropy, feature extraction, motor imagery electroencephalography, weight factors

## Abstract

Motor Imagery Electroencephalography (MI-EEG) has shown good prospects in neurorehabilitation, and the entropy-based nonlinear dynamic methods have been successfully applied to feature extraction of MI-EEG. Especially based on Multiscale Fuzzy Entropy (MFE), the fuzzy entropies of the *τ* coarse-grained sequences in *τ* scale are calculated and averaged to develop the Composite MFE (CMFE) with more feature information. However, the coarse-grained process fails to match the nonstationary characteristic of MI-EEG by a mean filtering algorithm. In this paper, CMFE is improved by assigning the different weight factors to the different sample points in the coarse-grained process, i.e., using the weighted mean filters instead of the original mean filters, which is conductive to signal filtering and feature extraction, and the resulting personalized Weighted CMFE (WCMFE) is more suitable to represent the nonstationary MI-EEG for different subjects. All the WCMFEs of multi-channel MI-EEG are fused in serial to construct the feature vector, which is evaluated by a back-propagation neural network. Based on a public dataset, extensive experiments are conducted, yielding a relatively higher classification accuracy by WCMFE, and the statistical significance is examined by two-sample *t*-test. The results suggest that WCMFE is superior to the other entropy-based and traditional feature extraction methods.

## 1. Introduction

In response to imaginary movements, the brain cortex produces a corresponding Motor Imagery Electroencephalography (MI-EEG) with a rhythmic activity. The MI-EEG-based Brain–Computer Interface (BCI) technology appeals to patients with neurological disabilities, such as stroke; it can help them with neurorehabilitation to restore more effective motion control [1,2]. Due to the sensitivity to noise, time-varying and fuzziness of MI-EEG, its feature extraction has become an important issue in BCI-based rehabilitation engineering.

At present, many feature extraction methods have been developed in time, frequency, time–frequency and spatial domains. The Autoregressive (AR) model is a classical feature extraction method in time domain. EEG signals are modeled by AR model and AR coefficients act as the features of EEG. This method can reflect the time-varying property of EEG, but it is sensitive to the data length [3]. The conventional time–frequency methods include Hilbert Huang Transform (HHT) [4,5], Empirical Mode Decomposition (EMD), Multivariate Empirical Mode Decomposition (MEMD) and Short Time Fourier Transform (STFT) [6,7,8], Discrete Wavelet Transform (DWT) based on Wavelet Transform (WT) [9,10,11] and Wavelet Packet Transform (WPT) [12], etc., in which the power spectral density, marginal energy spectrum, wavelet coefficients, and Wavelet Packet Entropy (WPE) are the commonly used features. The Common Spatial Pattern (CSP) is a classical spatial domain method, and it deals with two-class conditions in which the transformed projection signals are used as features [13,14]. Independent Component Analysis (ICA) combined with WT is also a classical method for MI classification; these traditional methods have exhibited the perfect performance in application of MI-EEG analysis, and their combination is also employed to realize the multi-domain feature fusion [15]. 

With the development of nonlinear dynamics, the entropy-based methods provide good alternatives to extract features of EEG and they can quantify the complexity and the irregularity of EEG signals as well. Approximate Entropy (ApEn) and Sample Entropy (SampEn) were first utilized in the field of biomedical signals processing [16,17,18]. Notably, the usage of Heaviside function yields the heavy dependence on the parameters’ selection and the discontinuity of entropies [19]. Focusing on the problem, Fuzzy Entropy (FE) was proposed by replacing the Heaviside function with fuzzy membership function [20]. It not only tackles the problem of entropy mutation, but also has good robustness to noise. In addition, Permutation Entropy (PE) was also applied to the analysis of EEG signals [21,22]. However, the referred methods are based on the single-scale and they may yield contradictory and misleading results. To discover deeper information, Multiscale Sample Entropy, (MSE) [23,24], Multiscale Permutation Entropy (MPE) [25,26,27] and Multiscale Fuzzy Entropy (MFE) based on Fuzzy Entropy (FE) [28,29,30], were also proposed, and MFE was further improved with the parameters’ independent optimization strategy, producing the improved MFE (IMFE) [31]. The previous studies have shown effectiveness of multiscale entropy methods. It is regrettable that the entropy mutation is inevitable when these methods are employed to short time series. So, Composite Multiscale Fuzzy Entropy (CMFE) was put forward [32,33], in which the *τ* coarse-grained series could be obtained by using moving average for a scale *τ*, and their FEs were averaged to form CMFE. It is noticeable that the coarse-grained procedure in CMFE amounts to mean filtering from the viewpoint of signal processing, and same weight factors are given to different sample points. It is unreasonable for nonstationary MI-EEG signals. In this paper, a Weighted CMFE method (WCMFE) is developed by introducing the weight factors in coarse-grained process, namely, assigning the different weight factors to different sample points, to change the coarse-grained series and their CMFE, which is more beneficial to the feature expression of non-stationary MI-EEG. Then, WCMFE is used to extract the nonlinear dynamic features of MI-EEG, and a Back-Propagation (BP) neural network is applied to classify MI tasks. It is further confirmed that WCMFE is superior to the other entropy-based methods and traditional feature extraction methods through experiments. 

This paper is organized into five sections. In Section 2, WCMFE is introduced to extract features of MI-EEG. Experimental research is performed on a publicly available dataset in Section 3. Section 4 makes the discussion and the conclusions are drawn in the final section.

## 2. Feature Extraction Based on WCMFE

CMFE has better stability and consistency than MFE and other entropy-based methods. It benefits from the multiple information of different coarse-grained sequences at the same scale factor [34]. Each coarse-grained time series of CMFE is approximately regarded as the arithmetic mean filter [35], which is helpful to eliminate general random disturbance and make the signal smoother. Nevertheless, this method fails to match the time-varying MI-EEG signals. It is necessary to change the weight factors in the coarse-grained procedure. In fact, the arithmetic mean filters will be replaced with weighted mean filters from the perspective of signal processing [36,37]. In the following, a Weighted CMFE (WCMFE) is developed and used for extracting the nonlinear features of MI-EEG. The detained process is described as follows.

### 2.1. Preprocessing of MI-EEG Times Series

Suppose that $X_{0}^{M}={\left[x^{M}(1),x^{M}(2),\cdots ,x^{M}(j),\cdots x^{M}(N)\right]}^{T}\in R^{N\times 1}$ with length *N* is the *M-*th channel MI-EEG signal of a trial, where $M=1,2,\cdots ,n_{C}$, $n_{C}$ is the total number of channels. By analyzing the impact of scale factor *τ* on CMFE of MI-EEG, the suitable value of *τ* can be selected. Then, the coarse-grained sequences and the corresponding CMFE in the scale factor are calculated, and the optimal time interval is determined according to the maximum difference of CMFE between two motor imagery tasks. So, the MI-EEG signal is rewritten as $X_{1}^{M}={\left[x^{M}(b),\cdots ,x^{M}(d)\right]}^{T}\in R^{H\times 1}$, where H=d−b+1, b and d represent the serial numbers of the start point and the end point in the optimal interval, respectively. 

### 2.2. Coarse-Graining for WCMFE 

In the coarse-grained process, the weight factors $A_{\tau ,h}=\left\{A_{\tau ,h}\left(1\right),A_{\tau ,h}\left(2\right),\cdots ,A_{\tau ,h}\left(\tau \right)\right\}\in R^{1\times \tau }$ are introduced, where $\sum_{k=1}^{\tau }A_{\tau ,h}\left(k\right)=1$, *τ* is the scale factor, and h∈{1,2,3,4} represents the selection mode of weight factors. Then, the *k-*th weighted coarse-grained sequence of $X_{1}^{M}$ in *τ* scale is described as $Y_{k,\tau }^{M}=\left\{y_{k,\tau }^{M}(j),1\leq k\leq \tau ,1\leq j\leq N^{\prime }\right\}$, and $y_{k,\tau }^{M}\left(j\right)$ can be formulated as:

(1) $$y_{k,\tau }^{M}(j)=\frac{1}{\tau }\sum_{i=(j-1)\tau +k}^{j*\tau +k-1}A_{\tau ,h}(i)*X_{1}^{M}(i),1\leq i\leq H$$

where $N^{\prime }=\mathrm{int}[\frac{H}{\tau }]$.

### 2.3. The Calculation of WCMFE

The MFE of the coarse-grained sequence $Y_{k,\tau }^{M}$ will be computed and noted as $MFE_{k,\tau }^{M}$, where $M=1,2,\cdots ,n_{C}$ and 1≤k≤τ. The algorithm steps are as follows:

(1) Given the embedding dimension *m*, the vectors $Y_{k,\tau }^{M}=\{y_{k,\tau }^{M}(i),y_{k,\tau }^{M}(i+1),\cdots ,y_{k,\tau }^{M}(i+m-1)\}-{\bar{y}}_{k,\tau }^{M}\left(i\right)$ are calculated, where $i=1,2,\cdots ,N^{\prime }-m+1$ and $\;{\bar{y}}_{k,\tau }^{M}\left(i\right)\;=\frac{1}{m}\sum_{l=0}^{m-1}y_{k,\tau }^{M}\left(i+l\right)$.
(2) For $i,j=1,2,\cdots ,N^{\prime }-m$ and i≠j, the distance between $Y_{k,\tau }^{M}(i)$ and $Y_{k,\tau }^{M}(j)$ is described as
  (2) $$d_{k,\tau }^{M,m}(i,j)=\underset{l\in [0,m-1]}{max}(|y_{k,\tau }^{M}(i+l)-{\bar{y}}_{k,\tau }^{M}(i)-(y_{k,\tau }^{M}(j+l)-{\bar{y}}_{k,\tau }^{M}(j))|),\;1\leq k\leq \tau$$
(3) For a given boundary gradient *n* and boundary width *r*, $\Phi_{k,\tau }^{M,m}\left(n,r\right)$ is calculated from Equation (3).
  (3) $$\Phi_{k,\tau }^{M,m}\left(n,r\right)=\frac{1}{N^{\prime }-m}\sum_{l=1}^{N^{\prime }-m}\left(\frac{1}{N^{\prime }-m-1}\sum_{i,j=1,j\neq i}^{N^{\prime }-m}\left(\exp(-\frac{{\left(d_{k,\tau }^{M,m}(i,j)\right)}^{n}}{r})\right)\right)$$
(4) Repeat the steps (1)–(3), $\Phi_{k,\tau }^{M,m+1}$ can be obtained. Then, $MFE_{k,\tau }^{M}$ is defined as
  (4) MFEk,τM(m,n,r,N′)=−ln(Φk,τM,m+1Φk,τM,m)

Hence, the WCMFE of the *M-*th channel MI-EEG can be calculated by averaging MFEs in *τ* scale:

(5) $$WCMFE_{\tau }^{M}(X_{1}^{M},A_{\tau ,h}^{},m,n,r)=\frac{1}{\tau }\sum_{k=1}^{\tau }MFE_{k,\tau }^{M}(m,n,r,N^{\prime })$$

### 2.4. Construction of Feature Vector

For *τ* scale, the WCMFEs of all the relevant channels can be expressed as

(6) $$F_{\tau }=[WCMFE_{\tau }^{1},WCMFE_{\tau }^{2},\cdots ,WCMFE_{\tau }^{n_{C}}]\in R^{1\times n_{C}}$$

where $1\leq \tau \leq \tau_{\max}$. The fusion feature vector of MI-EEG is defined as

(7) $$F=[F_{1},F_{2},\cdots ,F_{\tau_{\max}}]\in R^{1\times \left(n_{C}\times \tau_{\max}\right)}$$

## 3. Experimental Research

### 3.1. Data Source

**Figure 1:**
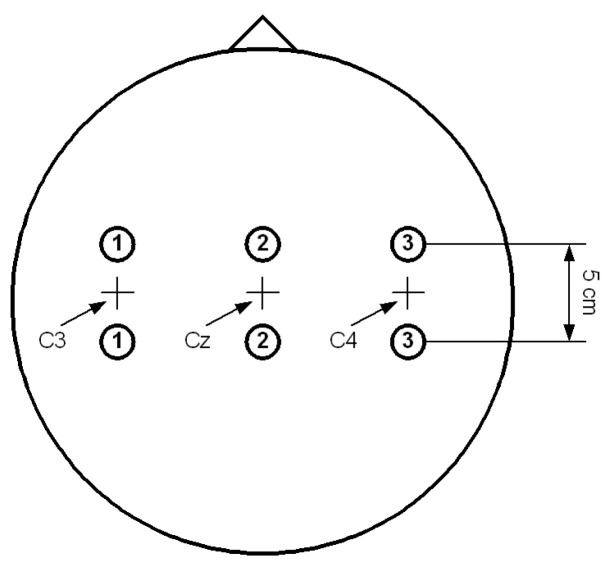
Electrode positions.

In this paper, the Data set III from BCI competition II is used to evaluate the superior performance of the proposed methods. This dataset was recorded from a normal subject (female, 25y) during a feedback session, and it was made up of the EEG signals about left–right-hand motor imagery. In the quiet and relaxed state, the corresponding imaginary tasks can be completed according to the screen prompt. Figure 1 displays the electrode positions. EEG signals were recorded on channels C3, Cz and C4 with sampling rate 128 Hz, and the signals were filtered from 0.5 to 30 Hz. There are 280 trials in the dataset. Similar to the training set, the test set was 140 trials in total, in which left and right hand motor imagery tasks were both performed 70 times. All trials were completed on the same day [38].

**Figure 2:**
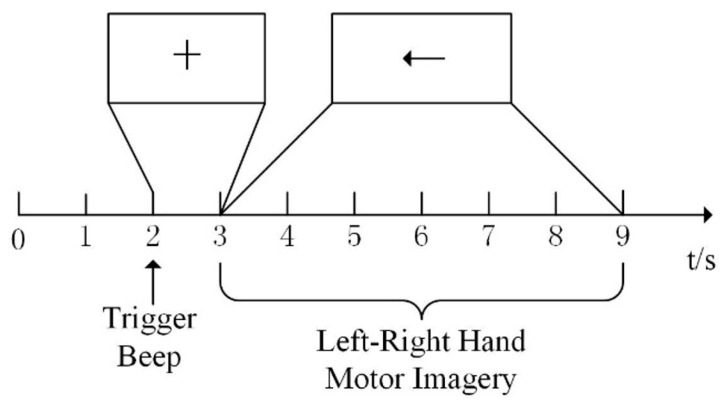
Timing scheme of Motor Imagery Electroencephalography (MI-EEG) collection.

As shown in Figure 2, a single test was 9s. In the first two seconds, the subject kept rest. When *t* = 2s, the screen displayed a “+” cursor and began with a trial sound signal. Between 3s and 9s, the subject proceeded with the corresponding imaginary task according to the direction of the arrow on the screen. 

### 3.2. Interval Selection of MI-EEG 

The data of the training set on channels C3 and C4 were applied to select the optimal interval. For left–right-hand imaginary movement, CMFE sequences of each trail in the training set on channels C3 and C4 were calculated, respectively. For the same task, CMFE sequences of 70 trials were superimposed and averaged by a sliding window with window length of 1s and step size of 1 sampling point to obtain the mean CMFE, where two channels of MI-EEG signals of 9s were considered in one trial, and the MI task was left or right hand motor imagery. Furthermore, the related parameters were selected as: m=2, n=2,r=0.15SD and τ=2. 

**Figure 3:**
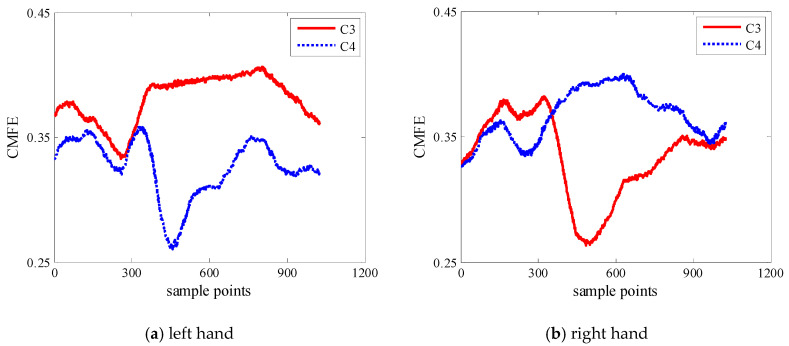
The mean Composite Multiscale Fuzzy Entropy (CMFE) time series curves of MI-EEG on channels C3 and C4 under different motor imagery tasks in condition of parameter settings: m=2,  n=2, r=0.15SD and  τ=2.

The mean CMFE time series curves are displayed in Figure 3. It can be seen that CMFE values on channel C3 increase gradually and on channel C4 decrease with the left-hand motor imagery. However, it is opposite for the right-hand motor imagery. It is concerned with the Event-Related Desynchronization (ERD)/Event-Related Synchronization (ERS) phenomenon. Moreover, within the sampling interval of [450, 900], the changes of mean CMFE on channels C3 and C4 are prominent and the difference is the most obvious, so the sampling interval in [450, 900] is chosen for subsequent experimental study. 

### 3.3. Comparative Study of WCMFE and CMFE

#### 3.3.1. Selection of Weight Factors

**Figure 4:**
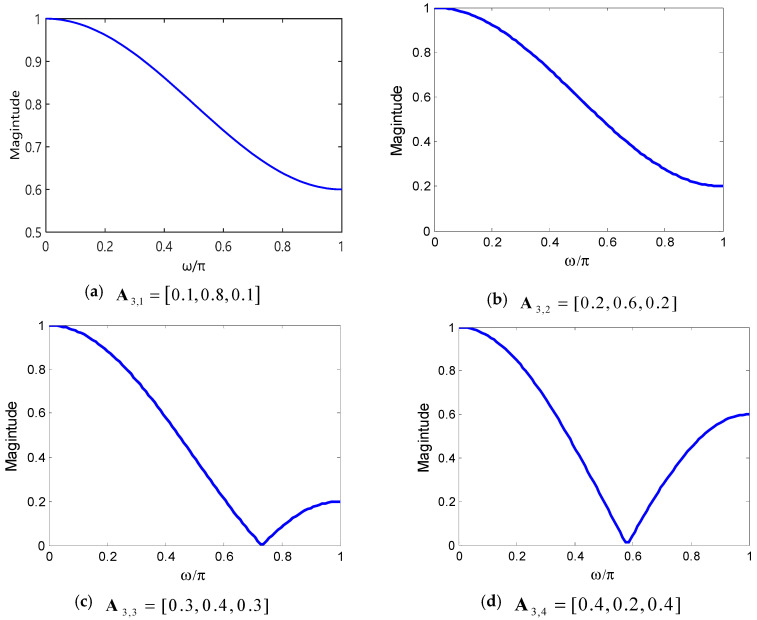
The spectrums of the three-point weighted mean filters with different weight factors.

It is necessary to select the appropriate weight factors for estimating the WCMFE of MI-EEG. Different weight factors of coarse-grained series on WCMFE will change the performance of the filter and affect the classification results successively. The three-point weighted mean filters with different weight factors (a) $A_{3,1}=\left[0.1,0.8,0.1\right]$, (b) $A_{3,2}=[0.2,0.6,0.2]$, (c) $A_{3,3}=[0.3,0.4,0.3]$ and (d) $A_{3,4}=[0.4,0.2,0.4]$ are constituted in Figure 4. It shows the variation of amplitude with normalized frequency ωπ. From the spectrums, the weighted mean filter has a low-pass characteristic and it can restrain high frequency components of the original signal. Meanwhile, the selection of weight factors will change the cut-off frequency and the spectrums as well. 

Linear phase Finite Impulse Response (FIR) filter can process data without phase distortion and it has been widely used in speech signal processing, adaptive processing and other aspects [39]. Its unit impulse response has symmetry property. According to the basis, the weight factors are only selected by the coarse-grained procedure and the rules are as follows: Define the weight factors as $A_{\tau ,h}=[\frac{h}{10},\frac{5-h}{5\times (\tau -2)},\cdots ,\frac{5-h}{5\times (\tau -2)},\frac{h}{10}]$, where $\sum_{k=1}^{\tau }A_{\tau ,h}\left(k\right)=1$, τ>2 and 1≤h≤4. Specifically, ${Α}_{\tau ,1}$ means that the first and the last points remain equal and are set as 0.1, $A_{\tau ,2}$ means the start and the end points are both set as 0.2 and so on. In addition, the middle τ−2 points of $A_{\tau ,h}$ remain equal and the sum of the weight factors is 1. When the other parameters are fixed, select different weight factors to calculate WCMFEs in multiscale and input to BP neural network to classification. The appropriate weight factors can be selected according to the recognition rate. 

**Figure 5:**
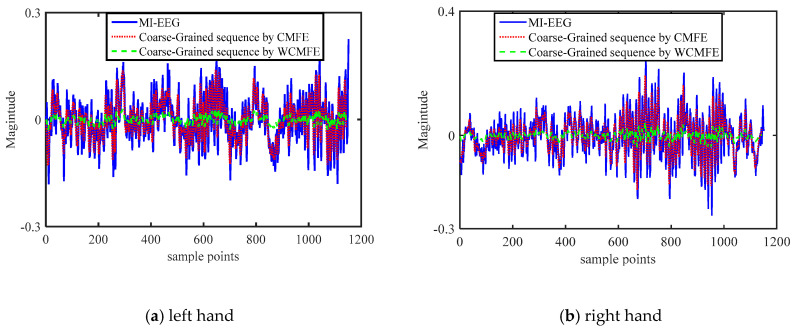
Comparison of the original MI-EEG and the coarse-grained sequences by using CMFE and Weighted CMFE (WCMFE) under different motor imagery tasks.

The coarse-grained process of CMFE can be regarded as the arithmetic mean filter, which can eliminate the noise to a certain extent and the calculation is simple. In order to detect the filter effect, a comparative experiment of the coarse-grained sequences using WCMFE, CMFE and original MI-EEG on channel C3 with motor imaginary tasks was carried out. Moving average was realized by a sliding window with sample interval of one. In Figure 5, there is no doubt that all of the coarse-grained sequence curves follow the trend of the original MI-EEG and weaken the influence due to noise or exceptional circumstances of MI-EEG to a certain degree. Further, they play the role of smoothing. The coarse-grained curves by CMFE have larger fluctuation and more dispersed points than WCMFE, which will yield misclassification in recognition and the poor classification accuracy. In contrast, the filtered MI-EEG by WCMFE is smoother and has lesser short-term variations. It is advantageous to correctly distinguish the different motor imaginary tasks.

**Figure 6:**
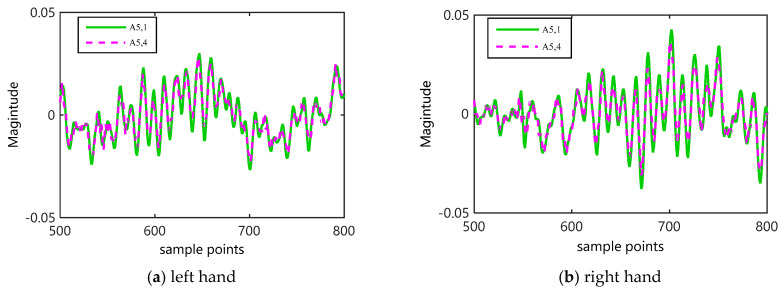
For τ=5, the effect of weight factors on coarse-grained sequences under different motor imagery tasks.

To further explain the effectiveness of weight factors in WCMFE, a comparison of the coarse-grained sequences on channel C3 was performed with a sliding window. In this case, the sampling interval was intercepted into [500, 800] to better display the difference between two motor imagery tasks, and the weight factors of $A_{5,1}=[\frac{1}{10},\frac{4}{15},\frac{4}{15},\frac{4}{15},\frac{1}{10}]$ and $A_{5,4}=[\frac{2}{5},\frac{1}{15},\frac{1}{15},\frac{1}{15},\frac{2}{5}]$ were selected. In Figure 6, differences of the coarse-grained sequences after these two weighted methods are displayed. In general, the variation trend of coarse-grained sequences produced by the two types of weighting factors is consistent, and the stability of the coarse-grained sequences with A_5,4_ is better than that with A_5,1_.

**Figure 7:**
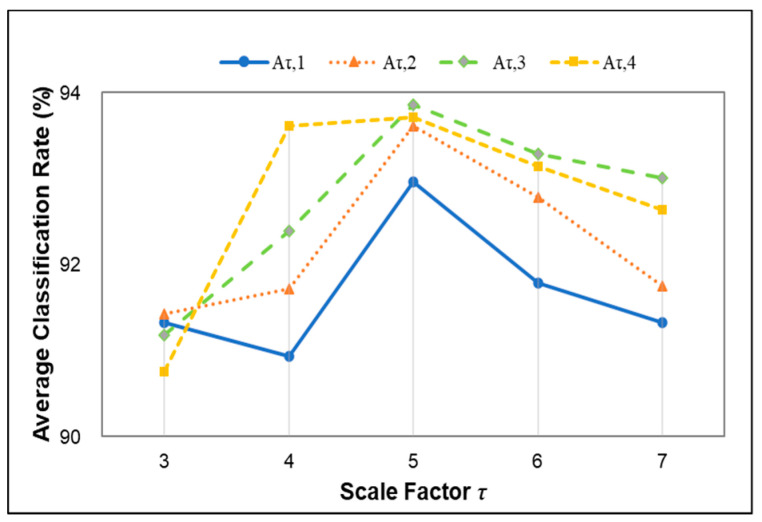
The average classification rate of different weight factors at multiple scales.

To acquire better classification results, more appropriate weight factors were selected through experiments. For τ=3∼7,m=2,n=2,r=0.15SD, the weight factors were set as $A_{\tau ,1},A_{\tau ,2},A_{\tau ,3},A_{\tau ,4}$ to compare. The experimental results are shown in Figure 7. It illustrates that multiple weight factors yield different recognition results. For *τ* = 4, the most obvious difference in classification results is between $A_{4,1}$ and $A_{4,4}$, and it is almost up to 2%. It implies that the appropriate weight factors are very important to obtain a better classification result; the four curves of the recognition results are basically Gaussian distribution with the increase in the scale factors. Further, from *τ = *5 to 7, $A_{\tau ,3}\geq A_{\tau ,4}\geq A_{\tau ,2}\geq A_{\tau ,1}$ are displayed in order of classification results. When *τ* = 5, all of the four groups’ classification results are the best and $A_{5,3}$ has the highest recognition rate, suggesting that $A_{\tau ,3}$ is more suitable for MI-EEG in this study.

#### 3.3.2. Selection of Scale Factor 

**Figure 8:**
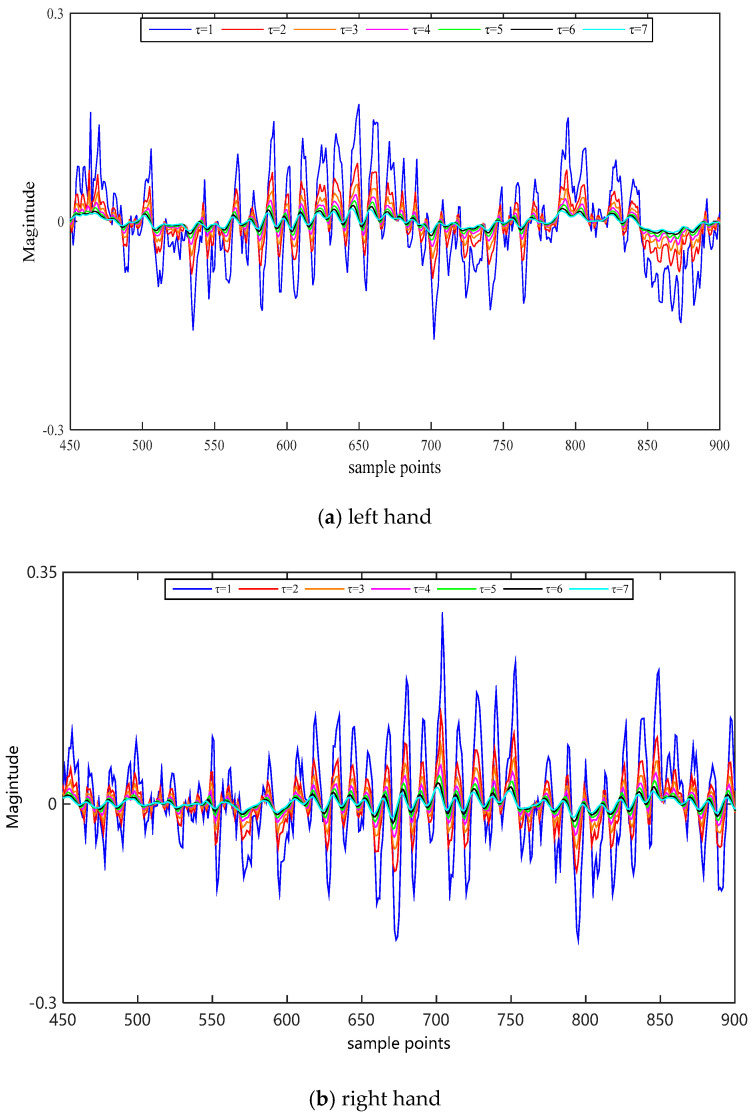
For τ=1∼7, the coarse-grained sequences obtained by WCMFE on channel C3 under different motor imagery tasks.

Selection of scale factor needs to be taken seriously. Different scale factors will change the information we obtain and affect the recognition results of MI-EEG in turn. Note that we only do the coarse-grained procedure to select the scale factor *τ* and the detailed rules can be summarized as follows: There is no coarse-grained operation when *τ*=1, which means the original MI-EEG. At the sampling interval [450, 900], two trials on channel C3 were selected randomly for imaging left–right hands movement. For the weight factor $A_{\tau ,3}$, the MI-EEG signals were filtered by the coarse-grained process of WCMFE on different scales. The filtered results are displayed in Figure 8. At multiscale, the coarse-grained sequences fluctuate with the trend of the original MI-EEG, but the fluctuation is smaller. Additionally, with the increase in the scale factor, the smoothness of the filtered MI-EEG is better. At the expense of it, the differences of the curves after filtered become smaller and smaller. Therefore, it has the maximum scale factor of 7 in this study.

#### 3.3.3. Selection of Parameters in FE

It can be concluded from Equation (5) that, except for the original sequence length *N*, the weight factors and the scale factor *τ*, the calculation of WCMFE is also related to the embedding dimension *m*, the boundary width *r* and the boundary gradient *n*. In this section, we will determine the selection of *m*, *n*, and *r* through experiments to optimize the recognition performance of MI-EEG. 

The detailed selection rules are as follows: The parameters *m* and *n* are fixed to calculate the mean and standard deviation of WCMFE with parameter *r* for two-class imaginary tasks, respectively. Analogously, we can obtain the mean and standard deviation of WCMFE with parameter *m* or *n*, respectively. 

**Figure 9:**
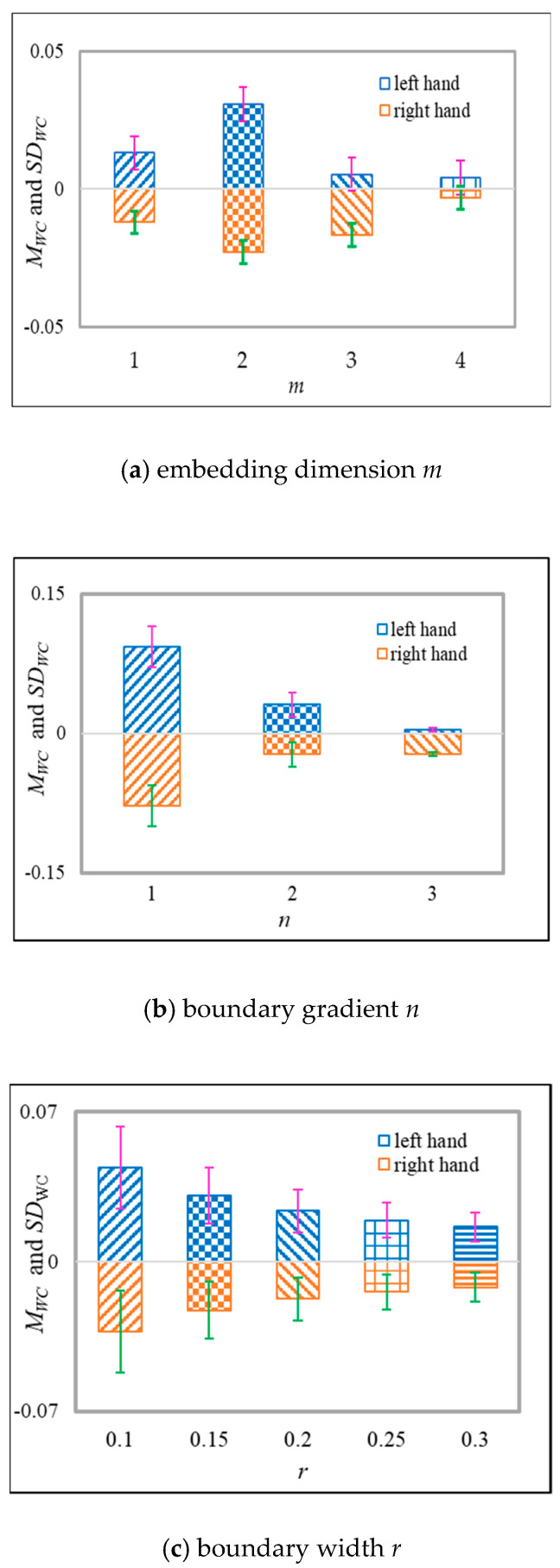
For τ=3, the variation of mean and standard deviation of $D_{WC}$ with the parameter *m*, *n* or *r*.

At the sampling interval of [450, 900], training samples of 140 trials were selected to calculate the mean WCMFE on channels C3 and C4, respectively. Then, the definition is as follows: $D_{WC}=WCMFE_{C3}-WCMFE_{C4}$, where $WCMFE_{C3}$ means the WCMFE of MI-EEG on channel C3, and $WCMFE_{C4}$ represents the WCMFE of MI-EEG on channel C4. $M_{WC}$ and $SD_{WC}$ are the mean and the standard deviation of $D_{WC}$, respectively. For τ=3, they were drawn with the parameters *m*, *n*, and *r* in Figure 9. 

The bigger difference of $M_{WC}$ as possible and the smaller values of $SD_{WC}$ as possible are the statistical basis of selecting the parameters of *m*, *n* and *r* for better discrimination of two motor imagery tasks. In Figure 9a, it illustrates the changes of $M_{WC}$ and $SD_{WC}$ with the embedding dimension *m* when *τ =* 3, *n* = 2 and *r* = 0.15*SD*. The difference of $M_{WC}$ between the two motor imagery tasks is the most obvious at *m* = 2 and it is the most beneficial to classification. Therefore, *m* = 2 is taken into consideration. The parameter *n* determines the boundary gradient of the similar tolerance in the process of fuzzy calculation. The variations of $M_{WC}$ and $SD_{WC}$ with *n* are shown in Figure 9b when *τ =* 3, *m* = 2 and *r* = 0.15*SD*. The larger *n* is, the larger the gradient will be, and the more information will be lost [40]. On the contrary, $SD_{WC}$ is the largest when *n* is set as 1. The effects of $D_{WC}$ and $SD_{WC}$ on classification are synthesized, a smaller *n* is selected to 2 in this paper. Similarity tolerance *r* mainly controls the similarity of template matching [37]. Figure 9c displays the changes of $M_{WC}$ and $SD_{WC}$ with the boundary width *r* when *τ =* 3, *m* = 2 and *n* = 2. Seen from Figure 9c, with the increase in *r*, the distinction between the left–right hand motor imagery is going down as well as the values of $D_{WC}$. In general, more difficult the matching of templates will be with the increase in *r*. Nevertheless, $SD_{WC}$ increases with the decrease in *r*; it is also harmful to classify and leads to increased sensitivity to noise. Therefore, we will select *r* = 0.15 in this paper. 

#### 3.3.4. Comparison of WCMFE and CMFE

**Figure 10:**
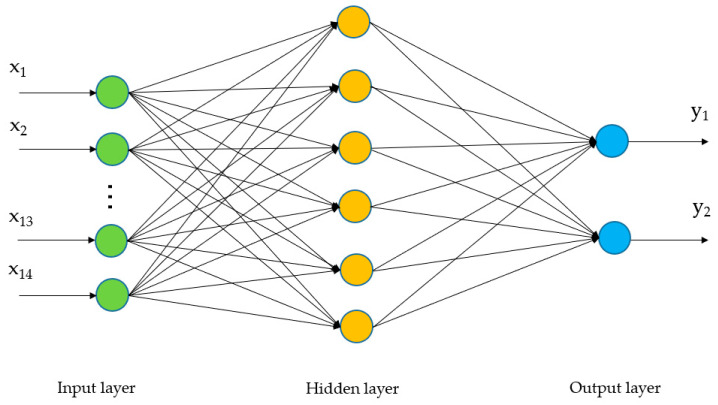
The structure of the Back-Propagation (BP) neural network.

Traditional classification algorithms mainly include linear discriminant analysis, SVM, logistic regression and so on. As a complex nonlinear problem, EEG should be considered from the nonlinear perspective in feature extraction and classification algorithms. The Back-Propagation (BP) neural network is a multi-layer feedforward network trained according to the error back-propagation algorithm. Without limiting the number of hidden layer nodes, a Back-Propagation (BP) neural network with only one hidden layer can achieve arbitrary nonlinear mapping [41]. Therefore, the BP network can be used to learn the complex nonlinear problem of MI-EEG recognition, and it does not have any requirements or restrictions on the distribution of training sample data. To verify the effectiveness and separability of WCMFE for two kinds of MI tasks, the comparison between WCMFE and CMFE was conducted and BP neural network was utilized for classification. The BP neural network consists of an input layer, a hidden layer and an output layer. The neuron number of input layer equals to the dimension of feature vector, which is set to 14. The hidden layer is the encoders with six neurons while the output layer has two neurons. The activation functions of neurons are sigmoid functions for input layer and hidden layer, and they are pure linear functions for output layer. The mean squared error is used as the loss function to evaluate the performance during the training process. The structure diagram of the BP neural network is shown in Figure 10.

For the purpose of eliminating the contingency of the feature extraction, the average classification result can be taken as the average of 10 × 10-fold Cross Validation (CV) based on all 280 trails from the Data set III of BCI Competition II, which contains the training set and the test set; one half is left-hand motor imagery and the other half is right hand motor imagery. In addition, the experimental parameters were set as: $\tau_{\max}=7,\;m=2,n=2,r=0.15SD$ and $A_{\tau ,h}^{}=A_{\tau ,3}$. The classification results are displayed in Table 1. It is clear that although the top classification results of CMFE and WCMFE are equal, the average classification accuracy is slightly better than CMFE. 

In addition, computation cost is another important index of algorithm. CMFE and WCMFE are compared in the same software (Matlab 2015, Windows10) and hardware (a Hewlett-Packard computer, which is equipped with an Inter(R) Core (TM) i7-9700 CPU @ 3.00GHz, a NVIDIA GeForce RTX 2070 GPU) environment. The computation time of one trail is 6.454 ms for CMFE and 6.477 ms for WCMFE. The small difference is resulted from the coarse-grained process, which is a mean filtering in CMFE and a weighted mean filtering in WCMFE, namely, the weight factor is $A_{\tau ,h}=[1,1,\cdots ,1,1]\in R^{1\times \tau }$ in CMFE and $A_{\tau ,h}=[\frac{h}{10},\frac{5-h}{5\times (\tau -2)},\cdots ,\frac{5-h}{5\times (\tau -2)},\frac{h}{10}]\in R^{1\times \tau }$ in WCMFE for each scale $1\leq \tau \leq \tau_{\max}$,$\tau_{\max}=7$ and *h* = 1. So, τ multiplications are added in calculation of WCMFE for each scale factor τ. Even so, the computation costs of CMFE and WCMFE are very close because of the excellent performance of computer.

#### 3.3.5. Statistical Analysis

To further analyze the resulted classification difference from WCMFE and CMFE statistically, a two-sample *t*-test is devoted to detecting whether there is a significant difference when they are applied to extract features of MI-EEG. 

First, the Lilliefors test (lillietest) is used to verify whether the classification results produced by WCMFE and CMFE conform to the normal distribution. In our experiment, Population 1 and Population 2 represent the classification results of 10 × 10-fold CV corresponding to WCMFE and CMFE, respectively, and they all consist of 100 individuals. The results of the test are displayed in Table 2 where the output results include the Hypothesis test result *h*, which returned as a logical value, and the *p*-value, which returned as a scalar value in the range (0, 1). From Table 2, it is obtained that the output results of Population 1 are *h* = 0 and *p* = 0.50 > 0.05, which means that the hypothesis that Population 1 is a normal distribution is accepted, and the output results of Population 2 are *h* = 0 and *p* = 0.27 > 0.05, which means that the hypothesis that Population 2 is a normal distribution is accepted. So, the two populations all fit the normally distributed. 

Then, we test the homogeneity of equal pooled variance of populations by Test Grouped Data for Equal Variances and the null hypothesis of the test is that the variances of populations are equal. The results of the test are also shown in Table 2. Where the p-value of the Homogeneity test of variance is 0.09, which is greater than 0.05, it indicates that the null hypothesis that the variances of populations are equal is not rejected. Therefore, the results show that two populations are consistent with normal distribution with equal variance.

After the normal distribution and homogeneity of variance of two populations were examined, we would perform the two-sample t-test. Assume that two samples were chosen independently and randomly from the above-mentioned two normal populations with equal variances (namely, Population 1 and Population 2), and they had the same sample size, then the test statistic *t* could be calculated by Equation (8). Where ${\bar{M}}_{WCMFE}^{}$ and ${\bar{M}}_{CMFE}^{}$ are the mean values of the two samples, $n_{WCMFE}$ and $n_{CMFE}$ denote the sample size, and $S_{WCMFE}^{2}$ and $S_{CMFE}^{2}$ stand for the variance, respectively. Especially, $n_{WCMFE}$= $n_{CMFE}$=15.

(8) $$t=\frac{{\bar{M}}_{WCMFE}^{}-{\bar{M}}_{CMFE}^{}}{\sqrt{\frac{\left(n_{WCMFE}-1\right)S_{WCMFE}^{2}+\left(n_{CMFE}-1\right)S_{CMFE}^{2}}{n_{WCMFE}+n_{CMFE}-2}\left(\frac{1}{n_{WCMFE}}+\frac{1}{n_{CMFE}}\right)}}$$

Defined, the null hypothesis is $H_{0}$: the classification results of WCMFE and CMFE are derived from independent random samples from normal distributions with equal means; the alternative hypothesis is $H_{1}$: the results of WCMFE and CMFE are derived from populations with unequal means. The significance level can be set as α=0.05. The decision rule is to reject $H_{0}$, if:

(9) $$p=P\{t>t_{\alpha }(n-1)\}\leq 0.05$$

It can be obtained that the value of *t* is 3.01, and the corresponding value of *p* is 0.0055, which is less than 0.05. Hence, the null hypothesis $H_{0}$ is rejected at the 0.05 significance level. This indicates WCMFE outperforms CMFE in feature extraction of MI-EEG.

### 3.4. Comparison with Multiple Traditional Feature Extraction Methods Based on BP Neural Network

To illustrate the feasibility of WCMFE in extracting features of MI-EEG, the comparison experiment with DWT, WT+ICA, HHT and WPE in references [5,9,12,15] was carried out. It was executed based on the same dataset (Data set III from BCI Competition II) and BP neural network was the classifier. The classification results are shown in Table 3. It indicates that the classification result of integrating WCMFE with BP neural network is better than the referenced methods. WCMFE has the advantage to quantify the complexity and the irregularity of sequences than the traditional feature extraction methods. Additionally, weight factors of WCMFE reflect the important degree of different sample points and have better adaptability to nonlinear non-stationary signals.

### 3.5. Comparison of Multiple Entropy-Based Feature Extraction Methods 

**Figure 11:**
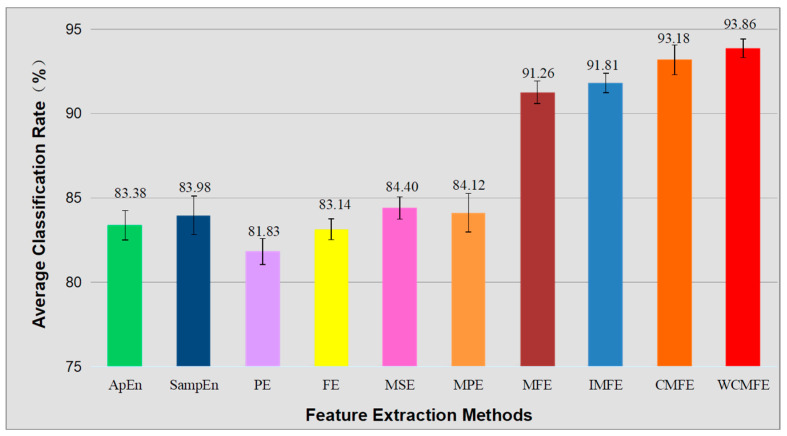
The average classification accuracies and standard deviations of 10 × 10-fold CV by using WCMFE and multiple nonlinear dynamic methods.

To verify the validity of WCMFE in extracting features of MI-EEG, some comparative experiments were performed based on various nonlinear dynamics methods in the same dataset. The average classification results are displayed in Figure 11. 

It is easy to see that ApEn and SampEn have poor classification performance. Due to the usage of Heaviside Function in the similarity measurement, it yields the mutation of entropy value. FE uses fuzzy function instead of Heaviside Function, which has better stability and consistency. However, this analysis ignores deeper feature information consistent with ApEn and SampEn. Thanks to the abundant characteristic information from multiple scales, we get better classification results when MSE, MPE, MFE and IMFE are designed to extract features of MI-EEG. Moreover, the CMFE method improves the performance of the coarse-grained sequences to overcome the drawbacks of the previous entropy methods, which has better stability for short time series. Due to the improved filter method of coarse-grained process, WCMFE enhances the recognition result. In addition, the standard deviation of 10 × 10-fold CV is smaller than the above entropy-based methods; it shows that WCMFE has better stability. Further, a two-sample *t*-test is designed to detect whether there is a significant difference between MFE and WCMFE or IMFE and WCMFE. The similar experiments were finished as in Section 3.3.5, and the values of *p* are both less than 0.01. It illustrates the superiority of WCMFE compared with MFE and IMFE in feature extraction of MI-EEG.

### 3.6. Comparison with Multiple Traditional Recognition Methods 

In this section, the comparison experiments with multiple recognition methods were carried out, including the other traditional recognition methods [5,6,7,8,10,11,12,15] and the top three recognition methods [42,43,44] based on the Data set III from BCI Competition II. Table 4 displays the detailed information. The combination of WCMF and BP achieves the highest recognition rate of 100%, and the average result of 10 × 10-fold CV is better than the best one in Data set III from BCI Competition II and the traditional recognition methods in references. It illustrates that WCMFE has better applicability to extract MI-EEG-related features, and it matches the BP neural network as well, which provides a new idea to extract features of MI-EEG signals.

## 4. Discussion

Entropy, as a measure of complexity, has received much attention and been developed well. Especially in consideration of the fuzzy, multiscale, nonstationary and individual difference characteristics of MI-EEG, a personalized WCMFE is proposed to explore its feature extraction problems. As an improved method of CMFE, the weight factors of the coarse-grained process in WCMFE were introduced to change the parameters and performance of filters, yielding the smoother, less overlapping and less fluctuation of filtered MI-EEG signals for left–right hand motor imagery tasks. It is helpful for signal filtering and feature extraction simultaneously, while the pure denoising technology cannot give consideration to feature extraction. Concerning this topic, successive studies were carried out. Based on the ERD/ERS phenomenon, the mean CMFE time series curves of MI-EEG on channels C3 and C4 were drawn in Figure 3 under different motor imagery tasks to determine the sampling interval for showing the best obvious difference, which helps find the time period that has class separability for a subject and will be used in the following experiments. Then, take the scale factor τ=3 as an example, the impact of weight factors on linear phase FIR filter performance was displayed in Figure 4, the symmetrical form of weight factors $A_{\tau ,h}$ was determined, and the resulting coarse-grained sequences were different from that of CMFE, as in Figure 5, which indicates that WCMFE can weaken the influence due to noise or exceptional circumstances of MI-EEG to a certain degree. To further demonstrate the effectiveness of different types of weight factors in WCMFE, the effects of weight factors on coarse-grained sequences are shown in Figure 6, which means that under the conditions of the same scale factor τ=5 and the different parameters h={1,4}, the changing trends of coarse-grained sequences are consistent, however, the vibration strengths are each different. Therefore, the multi types of weight factors $A_{\tau ,h}$(τ={3,4,5,6,7}, h={1,2,3,4}) were compared and the classification accuracies are shown in Figure 7, in which $A_{5,3}$ obtains the highest recognition rate and is more suitable for MI-EEG. Next, the parameters’ selection of WCMFE was discussed. When τ varied from 1 to 7 and the weight factor $A_{\tau ,3}$ was employed, the coarse-grained sequences over the best time period were demonstrated in Figure 8, where it can be seen that the change of coarse-grained sequences is getting weaker and weaker with the increasing of τ, and the difference between τ=6 and τ=7 is very small. Therefore, the maximum of scale factor $\tau_{\max}$ is set to 7. Furthermore, the embedding dimension *m*, boundary gradient *n* and boundary width *r* were studied through experiments in order to optimize the recognition performance of MI-EEG, and the results are shown in Figure 9 in the case of τ=3. In the following, a BP network, as a nonlinear classifier, was designed (as in Figure 10) to compare WCMFE and CMFE (see Table 1). After the Lilliefors test, to verify that the feature sample conforms to the normal distribution, shown in Table 2, a two-sample *t*-test was employed to detect whether there is a significant difference when WCMFE and CMFE are utilized to extract the nonlinear features of MI-EEG, WCMFE demonstrates the superiority of classification rate as well as the comparable computation cost. It is worth noting that WCMFE achieved a minor improvement of 0.68% in classification accuracy compared with CMFE; this might be because only the symmetrical form of weight factors $A_{\tau ,h}$ and the simple assignment of parameters were implemented. Further, the comparison experiments with traditional feature extraction methods, entropy-based nonlinear dynamic methods and multiple recognition methods in the references were carried out; for the details see Table 3 and Table 4, and Figure 11. It suggests that the results by WCMFE and BP neural network are better than the other methods, which indicates that the nonlinear features extracted by WCMFE is matched well with the nonlinear BP neural network classifier, and it is feasible and effective for the feature extraction of MI-EEG using WCMFE. It is also noticeable that the 10-fold cross validation is used for eliminating the contingency of feature extraction. 

However, we have to point out that the specific form and parameter values of weight factors in WCMFE are artificially set, and we have not a general method to set and obtain their optimal values; further research will continue with regard to the design and optimization of weight factors. In addition, we have only finished some research on a publicly available dataset, and two classes of MI-EEG were classified by using WCMFE. In the future, we will focus on the performance evaluation of WCMFE for multi-class motor imagery tasks and more subjects. 

## 5. Conclusions

Aiming at the non-stationary, multi-scale and individual difference characteristics of complex MI-EEG signals, a personalized WCMFE is developed by introducing weight factors in CMFE. Instead of the mean filters in CMFE, a weighted mean filter is applied to the original MI-EEG signal in each scale. This makes the filtered MI-EEG signal, namely, the coarse-grained sequences be coincident with the time-varying characteristic of MI-EEG and have less fluctuation than the original MI-EEG and the coarse-grained sequences by CMFE as well. It is helpful to objectively measure the complexity and represent the deeper nonlinear dynamic features in multiscale. The selection and optimization of the parameters in MFE are analyzed, and several setting modes of weight factors are given and discussed from the perspective of signal processing. The extensive comparative experiments are carried out on a publicly available dataset, and the relatively higher classification accuracy and the comparative computation cost show the effectiveness of WCMFE. The proposed WCMFE is an important supplement of Entropy theory, and it will promote the application of CMFE in time-varying signals, especially the biological signals such as Electrocardiographic (ECG) and Electromyographic (EMG). However, only the linear phase FIR filters were considered in our study and the parameters of weight factors were artificially set, this simplifies the design steps but limits the performance of WCMFE. How to design more reasonable filters to improve WCMFE is a potential problem.

## Figures and Tables

**Table 1:** Comparison of WCMFE and CMFE in the case of $\tau_{\max}=7,\;m=2,n=2,r=0.15SD$ and $A_{\tau ,h}=A_{\tau ,3}$.

Feature Extraction Method	Classification Method	Top Recognition Rate (%)	Average Recognition Rate with 10 × 10-fold CV (%)
CMFE	BP	100.00	93.18
WCMFE	BP	100.00	93.86

**Table 2:** Results of normal distribution test and homogeneity test of variance.

Type	Group	Count	Mean	*h*	*p*-Value
Normal distribution test	Population 1	100	93.86	0	0.50
	Population 2	100	93.18	0	0.27
Homogeneity test of variance	Pooled	200	93.52	-	0.09

**Table 3:** Comparison with Multiple Feature Extraction Methods.

Reference Number	Feature Extraction Method	Top Recognition Rate (%)	Average Recognition Rate with 10 × 10-fold CV (%)
[5]	HHT	87.14	-
[9]	DWT	92.40	-
[12]	WPE	88.57	-
[15]	WT+ICA	95.30	-
This paper	WCMFE	100.00	93.86

“+” means the combination of feature extraction methods or optimization of classifiers; “-” represents that the average recognition rate of ten times repetition of a 10-fold CV is not given in the reference.

**Table 4:** Comparison with multiple traditional recognition methods.

Reference Number	Feature Extraction Method	Classification Method	Top Classification Rate (%)	Average Classification Rate with 10 × 10-fold CV (%)
[5]	HHT	BP	87.14	-
[6]	EMD	POS+SVM	87.60	-
[7]	EMD	SVM	99.48	-
[7]	EMD+FE	KNN	99.39	-
[8]	MEMD+STFT	KNN	90.71	-
[10]	DWT+AR	LDA	90.00	-
[11]	DWT+FE	SVM	98.44	-
[12]	WPE	BP	88.57	-
[15]	CSP	SVM	82.86	-
[42]	WT	Bayes	89.29	-
[43]	ERD	LDA	86.43	-
[44]	AR	LDA	84.29	-
This paper	WCMFE	BP	100.00	93.86
